# Pain care coordination in the Veterans Health Administration: a rapid qualitative analysis of care coordinator experiences

**DOI:** 10.3389/frhs.2026.1856038

**Published:** 2026-06-17

**Authors:** Danielle Wesolowicz, Amanda Cary, Diana Higgins, Rebecca Kinney, Aimee Kroll-Desrosiers, Sara Edmond

**Affiliations:** 1 VA Connecticut Healthcare System, West Haven, CT, United States; 2 Yale School of Medicine, New Haven, CT, United States; 3 Veterans Integrated Service Network 8 Clinical Resource Hub, Tampa, FL, United States; 4 VA Durham Healthcare System, Durham, NC, United States; 5 Boston University Chobanian & Avedisian School of Medicine, Boston, MA, United States; 6 VA Central Western Massachusetts Healthcare System, Leeds, MA, United States; 7 University of Massachusetts Medical School, Worcester, MA, United States

**Keywords:** care coordination, care integration, pain management, pain management team, veterans

## Abstract

Pain care coordination is an integral part of care for veterans receiving pain treatment at the Veterans Health Administration (VHA). Pain care coordinators (PCCs) work to integrate care activities across the pain management spectrum. However, little is known about the make-up of the PCC role in contrast to other VHA care coordination positions, given the implementation of new VHA pain management roles over the past few years. The objective of the current study was to evaluate PCC perspectives of their role within pain management teams and to identify barriers and recommendations related to performing responsibilities as a PCC. Interviews were conducted with 11 PCCs across 10 VHA facilities, nationwide. Themes were identified using rapid qualitative analysis. PCCs represented a diverse group of providers, including nurses, social workers, pharmacists, and peer support specialists. Three major themes were identified: 1) diverse ways in which PCCs operated in their role, 2) PCC experiences working as a member of a pain management team, and 3) their perspectives on working in a shifting pain management landscape. PCCs can serve a unique and valuable role within pain management settings. Efforts to improve the implementation of care coordination on pain management teams should include PCC role clarification, training, and system-wide efforts to reduce barriers to patient-centered care.

## 1 Introduction

Veterans experience chronic pain at a higher rate than civilians (1) and pain prevalence among veterans is increasing (2). Further, veterans are more likely to experience high-impact chronic pain (i.e., pain that significantly limits daily activities), compared to the general population (3). Veterans have several potential options available for pain management within the Veterans Health Administration (VHA) healthcare system, with comprehensive and multimodal treatment strategies increasingly encouraged by the VHA over the past two decades (4, 5). In 2009, the VHA introduced the Stepped Care Model of Pain Management, which provided a framework for effective treatment of pain by emphasizing an individualized approach to pain management informed by the veteran (6). In 2016, the Comprehensive Addiction and Recovery Act (CARA) mandated that each VHA facility designate a pain management team comprised of an interdisciplinary team of providers (7). A fully staffed pain management team must include, at a minimum, members fulfilling all of the following roles: 1) a medical provider with pain expertise; 2) an addiction medicine provider with expertise to provide evaluation for Opioid Use Disorder and the ability to prescribe Medication-Assisted Treatment; 3) a behavioral medicine provider with availability of at least one evidence-based behavioral therapy for the management of pain; and 4) a rehabilitation medicine provider. In addition to this pain management team at all VHA facilities, full implementation of the stepped-care model was required in the CARA mandate.

To enable facilities’ ability to meet the CARA legislation mandates and ensure dedicated staffing for pain services and teams, the VHA Pain Management Opioid Safety and Prescription Drug Monitoring Program (PMOP) released an initiative to provide recurring funds for VHA regional systems of care and facilities to provide salary support for PMOP coordinators (provide administrative oversight to a facility’s pain management and opioid safety programs), points of contact (support the implementation of pain management initiatives and provide consultation on pain management practice, policy, and services), and primary care pain champions (work to promote best pain management practices in the primary care setting); the initiative additionally provided temporary funds for the expansion of veterans’ access to clinical pain care services that included nonpharmacological, holistic, and medication management-focused clinical programs in 2021.This funding initiative assures oversight, reporting, and coordination of pain care and opioid stewardship programs and initiatives.

Since the inception of this PMOP initiative, pain management teams have worked to develop their role in coordination of pain care in the VHA. Care coordination refers to a collaborative effort between healthcare providers and patients to facilitate the appropriate delivery of healthcare with a high degree of role specification, communication, and integration of care activities (8). Models of care coordination generally include a) targeting individuals most vulnerable to disconnected care, b) conducting assessments to identify problems and develop care plans, c) executing these care plans with a high degree of communication and coordination between care recipients and care providers, d) monitoring and adjusting care as needed, and e) evaluating health outcomes. Among the care providers themselves, care coordination also includes the development of collaborative, multidisciplinary teams that feature a case manager and/or patient navigator to help oversee and facilitate the care of high-risk patients (5).

Care coordinators are particularly well-suited to support improved care for high-risk and vulnerable patients, such as veterans with high-impact chronic pain (i.e., pain that affects the ability to participate in activities most days or every day) who experience significant psychosocial impairments and medical comorbidities. However, many facilities do not have a pain care coordinator, leading to gaps in pain care access and reduced outcomes for patients. Given the novel and evolving role of pain care coordinators (PCCs) within the VHA, the objective of this present work was to evaluate PCC perspectives of their role within pain management teams, and to identify barriers and facilitators that may exist related to their ability to perform their responsibilities.

## 2 Materials and methods

Potential interviewees were identified via a larger survey conducted with VHA facilities to understand pain management team staffing was used to identify facilities with a PCC serving on their pain management team. Survey respondents that self-identified as a PCC were invited to participate in a qualitative interview about their role on their respective pain management team. Potential participants were sent an e-mail invitation from the program evaluation qualitative team. Three follow-up e-mails were sent to help ensure that each care coordinator had the opportunity participate. In total, 20 care coordinators were identified and invited and of those, 11 (55%) completed a qualitative interview (see Table 1 for additional information).

**Table 1:** Characteristics of pain care coordinators .

Characteristic	Participated in Interview (n)	Total (n)
Clinician Type		
Registered Nurse	5	11
Licensed Practical Nurse	0	1
Peer Specialist	1	2
Pharmacists	2	2
Social Workers	3	4

A semi-structured interview guide (see Appendix A) was developed for the purposes of this project with content experts (e.g., pain management team clinicians, pain researchers) and qualitative experts; PMOP leadership also provided input on the interview guide. The interview guide explored how facility pain management teams function and included questions on typical patient flow, the roles and responsibilities of pain management team members both within and outside the pain team, perspectives on the team functioning, perceptions of reasons for the team being successful, and current team challenges and barriers. These semi-structured qualitative interviews were designed to last approximately 30 min. All participants were assured that individual responses would be held confidential and provided oral informed consent. Interviews were recorded and transcribed using Microsoft Teams. A rapid approach was utilized to analyze the data in effort to meet program office needs in a timely manner. Rapid Qualitative Analysis (RQA) is a validated, rigorous semi-structured method that is well suited for implementation-focused projects (9). RQA uses a mixed inductive/deductive approach to analysis whereby a deductive template is used to structure the analysis, but an inductive approach is used to identify specific findings that emerged from the data. The qualitative research team (AKD and RK) collaboratively created a summary template that corresponded to the semi-structured interview guide. Research team members listened to audio recordings of the interviews and summarized interview content in each of the domains; the first few summaries were reviewed between the interview team to establish consistency in summary style and content, and then the remaining interviews were independently summarized. Once all summaries were completed, summary points were transposed into a matrix (participant x domain) to aid in interpreting the findings. Analysis occurred with the research team and other authors (SE and AC); themes were identified using an iterative process in which the analysis team compared observations and reflections across the matrix. Once a consensus was reached on observations, cross-cutting themes were identified that reflected cross-cutting observations (9).

## 3 Results

Eleven interviews were conducted with PCCs during March and April 2023. Interviews were conducted by two of the authors who have expertise in qualitative methodology, including data collection and analysis (AKD and RK). The interviews were conducted by telephone and videoconferencing took place across ten facilities representing geographically distinct locations across the U.S. (Arizona, Connecticut, Hawaii, Kentucky, Massachusetts, Michigan, New Jersey, Oklahoma, Pennsylvania, West Virginia). VHA facilities included PCCs represented a diverse group of providers (Nurses: *n* = 5; Social Workers: *n* = 3; Pharmacists: *n* = 2; Peer Specialists: *n* = 1). Regarding facility complexity (a categorization of VHA facilities based on clinical and operational complexity), interviewees were employed at Highest Complexity (1a, *n* = 2), High Complexity (1b, *n* = 1), Mid-High Complexity (1c, *n* = 5), and Low Complexity (3, *n* = 3). After these 11 interviews the research team determined that no new insights were emerging from interview summaries and that data saturation was reached. Using the matrix analysis approach, interview summaries were organized by domain and participant to aid in analysis and interpretation, as described above. Three major themes were identified in the interview data as detailed below.

### 3.1 Theme 1: care coordination approaches and experiences vary by discipline

Pain management team care coordinators were asked about their role on their team, including their responsibilities in their coordinator position and what a typical day is like for them. Responses varied between disciplines, primarily in terms of what the PCCs viewed as their primary responsibilities. Social worker PCCs described their role as a combination of case management and therapeutic services to high-need veterans.

“I manage all the consults that we get for veterans who want to participate in the (pain management) program. I make sure they're scheduled and then I check-in on veterans, just making sure like they're meeting their goals and (asking about) what kind of problems they're having, things like that. (It is) kind of half case management, half therapy.”—Social Worker 1

Another social worker shared how their day-to-day responsibilities necessitate versatility:

“A typical day I might have some scheduled veterans for either the initial intake or for therapy services, or I might get a consult for the medical social work piece. Today it was, you know, trying to find transportation services for a veteran. That’s pretty typical of my day.you need to be kind of flexible.”—Social Worker 2

In contrast, Pharmacist PCCs described their roles as primarily administrative in nature, developing programs and services for the facility to adopt.

“When people ask me what I do (…) I basically tell them that I'm in charge of the program for pain management and opioid safety. (…) And so my role really is as a systems thinker to try to build the clinical programs and services available at our facility for pain management. I'm constantly moving from one project to another and I'm constantly asking people for updates as we continue to try to build our pain management services.”—Pharmacist 1

A second pharmacist shared their responsibilities related to education:

“I also do a lot of education on opioid safety initiatives and there’s a lot of dashboard monitoring… it is based on whatever issue is most pressing. We identify areas for improvement or other nationally driven PMOP focused initiatives. So, (for example) there’s the national initiative to require naloxone for certain high-risk groups, and then that becomes an action item that we send to our local leadership. It starts with the dashboard, looking up patients or providers or services that may require naloxone or could use some extra support for naloxone prescribing (and then) doing individual provider level and service level education.”—Pharmacist 2

Nurse PCCs described their positions as being closer to a more traditional nursing role, helping primary care providers with initial referrals and pain assessments. One nurse described her PCC role as “triaging patients, responding to phone calls, and responding to secure messages [from patients].” Another nurse elaborated:

“After we see (patients) that day, we come up with recommendations and they're handed a printed copy of all the recommendations. We'll place a lot of consults and help them make appointments. I put in a summary of the recommendations in their chart, and then I usually follow up like about a month later.”—Nurse 4

Finally, we spoke with a Peer Specialist who described their job as providing support to veterans as they engage in pain care:

“My job is engagement for the team. I am an engager in relationships. I build the relationships and the rapport to keep the veterans. A little bit of my duty is kind of a door greeter… I call (the veteran) ahead of time. When we can, we do a personal health inventory and share it with the pain management team before beforehand so that everybody’s a little bit more informed about the veteran.”—Peer Specialist

Without clear guidelines or expectations for the role, some interviewees reported they felt unsure how to navigate their coordinator role.

“A lot of the other VA’s have coordinators that aren't social workers, so that’s fine but we don't have any mentorship (…)I don't have any mandates in front of me. I've never been given anything that says from the national level, ‘It should look like this or flow like this’.”- Social Worker 3

Balancing the care coordination role with other professional identities was described as challenging:

“I think having the two different roles has been has been challenging…I mean because I've always been kind of a more on the therapy side of social work…but I mean always providing referrals and whatnot, but sometimes some of the medical, like traditional (primary care) stuff.”—Social Worker 2

### 3.2 Theme 2: team dynamics affect care coordination

Team dynamics and team communication style shaped how PCCs viewed their role on their pain management team. Telework can allow for flexibility in hiring pain management team clinicians and can address barriers related to clinic space limitations but it can lead to a sense of disconnect from their fellow team members.

“…our pain team is all hybrid, every provider is hybrid due to clinic space limitations (…) because our team is growing so quickly, not every provider can be here every day. So we have like a rotating schedule…”- Pharmacist 2

Even when physically located together, PCCs described communication challenges within their teams that limited their ability to operate effectively as a pain management team and help patients navigate pain care.

“The pain management team, the doctor recently left to go to another VA. And it was they were very siloed (…) I was trying to encourage us to have team meetings and whatnot, but that that never that never happened.”—Social Worker 2

Finally, some PCCs noted that the current structure of their teams made it challenging to optimize patient follow-up.

“We have a couple of doctors that will type up our recommendations and send it into a letter to the veteran … we need some kind of personal follow up from the pain clinic.”- Social Worker 3

“(…)I try really hard to keep track as much as I can on my end and make sure that that loop where they get called, who’s gonna call on how many times, you know, like that type of thing.”—Nurse 3

### 3.3 Theme 3: navigating care coordination in an evolving treatment landscape

A final theme that arose from pain care coordinator interviews was around challenges they faced navigating a changing pain management landscape and their suggestions for improvement. Coordinators discussed the importance of increasing access to different types of care and treatment modalities, including that of Whole Health (a holistic system of care within the VHA that includes health coaching and Complementary and Integrated Health Services like acupuncture, yoga, and tai chi/qi gong):

“Most VAs have a baseline set of programs and services, but some of these supplementary type or complementary type therapies like Whole Health vary from one facility to the next (…) if we're going to limit opioid use, we're going to have to provide patients with other alternatives for pain management.”—Pharmacist 2

“The Whole Health isn't good enough. It needs to be bolstered. It needs to be bigger. The culture change is way too slow, and in my opinion, they're not spending enough money on Whole Health because we're getting traction there. We need a bona fide dedicated instructor for Tai Chi and yoga, with multiple offerings during the week. They say that they're honoring Whole Health but then they don't actually put any resources behind it.”—Peer Specialist 1

However, a shift away from opioids and toward nonpharmacological pain management options, including Complementary and Integrative Health, has been challenging for some patients and providers to navigate. One PCC was concerned about pharmacological pain management access for their patients:

“It used to be where medications were overly prescribed, and now it’s almost like to the other extreme, where Veterans can hardly get anything prescribed and for such a limited time or they can end up getting cut off like what I think of as cold turkey. And to me that is very concerning. I feel like there’s a lot of scrutiny and Veterans are almost scared to be like ‘is it possible to get this medication?’ They feel like if they even ask for it that they're drug seeking when they're not.”—Social Worker 2

Care coordinators spoke about placing frequent referrals to community care (care provided outside of the VHA system), as many services were not offered within their facility; while interventional procedures are most commonly referred to community care, massage, chiropractic care, and acupuncture were also often discussed as services that may be referred to community care. Even among facilities that provided such services, increasing demand for those services leads to extended wait times and ultimately require community care referrals to keep pace.

“It’s due to wait times. I think our providers may be overwhelmed with demand as far as chronic pain management patients, and the most common reason that we're referring out would be for procedures due to wait time.”—Pharmacist 1

One nurse described their goals to educate VHA providers about treating the Veteran in a more comprehensive manner:

“I try to educate people in this establishment on the regular basis. Just because you work in primary care, audiology, neurology… guess what, the Veteran is a whole person, and they have challenges that may affect other disciplines. Just because you are looking at them for what you're dealing with them for, that doesn't mean that’s the case scenario.”—Nurse 5

## 4 Discussion

Care coordination efforts on pain management teams can help ensure all patients are able to engage in high quality, integrated pain care. In this qualitative study, PCCs had varying degrees of responsibilities and duties, typically dependent on their professional role (e.g., pharmacist, nurse, social worker). Providing education to patients and providers and engaging patients in goal-setting were described as key components of their roles as patient care coordinators. However, PCCs across disciplines shared common barriers of communication within their teams, difficulty navigating a changing pain management landscape, and promoting access to pain treatment options. These results reflect those of a recent qualitative study of pain Nurse Navigators that found while Nurse Navigators viewed care navigation as a critical component of their roles, barriers like limited specialist availability and access for rural patients could hinder effective care navigation (10).

Care coordination is frequently used throughout the VHA for a variety of chronic conditions (11). Specifically related to chronic pain, the Veterans Affairs Evidence-based Synthesis Program published an evidence brief on various models of multimodal pain care (12); among the analyzed models of care, four qualities were identified promoting guideline-concordant multimodal pain management: decision support, enhanced patient education and activation, increased access to a broader range of treatments, and additional care coordination resources. With the introduction of PMOP funding, many of these qualities have been implemented across the VHA. However, without clear national guidance regarding how PCCs should operate and other barriers, such as communication challenges, their potential impact for pain management may be limited.

Through this work, we identified several recommendations to facilitate successful implementation of care coordinators on pain management teams to maximize the benefit that PCC provide to the team and patients who are seeking pain care. Clear roles and responsibilities for the positions are needed, including clarity on the potential role of direct patient care among care coordinators. During the onboarding process, care coordinators may require training in several domains, such as best practices in chronic pain management, the structure and function of the pain management team, available resources at the facility, and information about the larger VHA system. Assessment of what types of care coordination activities are most appropriate and effective across settings could help optimize cost and effectiveness. To support improved referral processes, facility-wide training on pain management teams may also be necessary. Teams may feel better prepared to understand changes to the broader pain management landscape through ongoing training and the creation community of practices to help provide education and support to providers. An ongoing national, standardized effort to within the VHA is The Advancing Education for Pain Teams (ADEPT) Program that includes face-to-face and virtual sessions over the course of 6 months; the aims of this program are to enhance clinician skills in biopsychosocial pain assessment, communication, and promotion of evidence-based treatments. Finally, while care coordination support may enhance existing teams, other efforts are still needed to reduce barriers to patient care including addressing issues related to transportation, cost, and scheduling (13).

We acknowledge limitations with our research. A total of 11 care coordinators were interviewed, and it is possible that additional interviews would have uncovered supplementary themes. Further, because interviewees self-identified as a PCC there was a wider range of professions (i.e., pharmacists, social workers) included in our sample, our findings may reflect a broader conceptualization of care coordination activities (e.g., administrative) than would be included in other care coordination studies. Additionally, our use of rapid qualitative analysis was appropriate to deliver results in a timely manner to the sponsoring VHA program office, but it's possible that more traditional methods of qualitative analysis may have yielded more rich data. However, this work was strengthened by the diversity in geographical region and professional discipline of care coordinators interviewed.

## 5 Conclusion

In summary, this work examined the roles, experiences, and barriers to pain care coordination within VHA. The results of the current work offer an important look into implications and recommendations to strengthen current and future pain care coordination teams. These findings are critical as while care coordination has been studied, pain care coordination is a less investigated area although an integral aspect of serving veterans within VHA. The group of interviewed pain care coordinators came from a nationwide sample of VHA settings. They indicated diverse responsibilities within their pain management team roles particularly in their clinical duties, and highlighted opportunities for improvement around the areas of role clarification and team communication. This area of research has potential for significant implications as pain care coordination expands to meet veteran need throughout VHA.

## Data Availability

The datasets presented in this article are not readily available because given privacy concerns, data is not available by request. However, other materials like the interview guide can be shared upon reasonable request. Requests to access the datasets should be directed to danielle.wesolowicz@yale.edu.
