# Supplementary material for: Pain care coordination in the Veterans Health Administration: a rapid qualitative analysis of care coordinator experiences
Source: Front Health Serv. 2026 Jun 17;6:1856038. doi: 10.3389/frhs.2026.1856038 (PMC13319020; doi:10.3389/frhs.2026.1856038)
Supplement: Supplementary file 1 [file Datasheet1.docx]

**Appendix A: Pain Care Coordinator Interview Guide**

**Intro:**

As you likely know, the 2016 Comprehensive Addiction and Recovery Act (CARA) mandated that each VHA facility designate PMTs, interdisciplinary teams of providers that work together to conduct comprehensive pain assessments using a biopsychosocial framework and develop integrated, multimodal treatment plans for Veterans with chronic pain.

We know that these teams operate differently in each facility. Teams typically have a medical provider with expertise in pain, someone with expertise in addiction, someone with behavioral health training, and someone with a rehabilitation background. Some teams see patients together or are co-located, while others work more as a consultative network. Some teams primarily focus on assessment and treatment recommendations, while others follow patients longitudinally.

From our understanding, you are currently in a position where you coordinate care for Veterans in some capacity as part of an interdisciplinary pain management team at your facility. Is that correct?

1. Please describe the PMT at your facility. What role do you play? What responsibilities do you have at your facility? How long have you been serving in this role?
   1. Probe: What is your training background? What are your credentials? Did you have experience with pain management prior to being in this position?
   2. Probe: Are you the only PMT at your facility? Why or why not? Can you tell me more about the other pain teams at your facility?
   3. Probe: Are you the only person serving in a coordinator role? If so, how much of your time is devoted to work on the PMT?
   4. Probe: Can you tell me how the PMT functions at your facility in relation to other pain care?
2. Probe: What tasks do you find yourself doing in your coordinator role? How is this time spent? How do your day-to-day tasks differ from your written job duties? What kinds of tasks take up a lot of time during your day and how is this managed? (e.g., a constant stream of patients/patient questions)
   1. Probe: Do competing priorities impact your role on the PMT? Is your role protected or a collateral duty? If yes, what percentage is protected? If you have protected time, do the responsibilities ever exceed your bandwidth? How so?
   2. Probe: How is your position funded? Do you have expectations for billing that supports your position? When you document do you code for things that gets your facility workload credit?
3. What is the process for the PMT providing care to a new patient? Is there a written protocol? What specific information is given to the Veteran about the PMT?
   1. Probe: How are Veterans screened to determine who would benefit for care coordination? Are Veterans screened to determine who would benefit from related care, such as mental health? Is there an electronic health record review process in place? How are patient need levels classified (e.g., basic, moderate needs)?
   2. Probe: How do you get referrals to your pain team? Are referrals tracked? If so, who is responsible for that tracking?
   3. Probe: Can you describe the typical patient flow*,* the referral pathway to and from primary care at your facility? What is that flow like? What role do you play in managing this flow?
   4. Probe: Are there specific criteria for which patients are seen by the PMT (e.g., does your team only see patients prescribed opioids)? Do you only see patients if they’ve completed other treatments in the past?
   5. Probe: How often are you working directly with Veterans, answering questions, helping them understand their care, etc.?
4. What are the tracking systems for following a Veteran’s medical care while they are seen by the PMT? By what mechanisms does this occur? How are you involved in the coordination of these processes?
5. Please describe your interactions with other VHA staff regarding the care provided by the PMT. What type of communication do you have with these staff members? When does this occur? What information do you share?
   1. Probe: How do you communicate with the team? Do you have team meetings or use another form of communication often? Who is responsible for the organization/scheduling of these meetings?
   2. Probe: How do you communicate or coordinate with other pain services (i.e., pain and non-pain services such as mental health, primary care)? Who is the primary contact for a new patient? How do you help your patients access those services?
   3. Probe: Can you describe how the PMT communicates with other service lines at your facility? For example, other pain services as well as non-pain services such as mental health and primary care. What is your role in these communications as a coordinator? What role do you play in talking to people outside of the team in these different settings?
   4. Probe: Could you tell me about how transferring prescribing back to primary care works? How do you ensure continuity of care? Is there continued follow-up from the pain clinic at larger intervals?
6. What do you observe or hear from Veterans about their pain health care needs? Who typically addresses those needs?
   1. Probe: At your facility, what challenges have been identified in meeting the needs of patients with complex chronic pain? In what ways do you think the PMT could be more helpful to primary care in this realm?
   2. Probe: Are you aware of metrics that are used to measure successful pain treatment for Veterans? How about metrics related to reducing unnecessary tests?
   3. Probe: Do Veterans provide any direct feedback about the care they receive?
7. How supportive has your facility been in helping your role as a coordinator be developed within your PMT? What has helped or hindered this process?
   1. Probe: Do you feel as that there is enough leadership and support for your coordination role on the PMT?
   2. Probe: How does your supervisor or facility leadership help support you with difficult situations? For example, this may include dealing with a difficult patient or resolving conflicts with colleagues about patient care.
   3. Probe: Are there disparities between what the PMT expects of you as a coordinator and what your service line expects of you?
8. How do you think specific organizational characteristics (size, location, health resources, affiliation with academic medical center) has impacted your PMT?

Now I’d like to ask you about Community Care, specifically whether care in the community impacts your work on the PMT when Veterans are referred out. Could you tell me about pain services that Veterans are often referred out into the community for their care?

Probe: What role (if any) do you play in this process? If not you, who is the person who leads this effort (i.e., what discipline are they in)?

Probe: Is there any tracking or reporting back to the PMT?

- 1. Probe: What improvements could be made regarding the types of information you get back from community providers?

1. What additional services do you think the VA might need to provide to improve pain care for Veterans? Are there any additional types of care coordination that Veterans need? How can services or policies be improved to enhance pain care coordination at your facility?
   1. Probe: What are the strengths of your PMT? How does your coordination position impact these strengths?
   2. Probe: How can your PMT improve? Are there any areas of weakness or barriers to providing care (e.g., communication or staffing issues)? Lack of certain resources? What resources would help your most in your day-to-day position?
   3. Probe: What are your PMT’s biggest struggles? How can these needs be addressed?
